# Impact of Severe Postoperative Complications and P-POSSUM Score on Oncological Outcomes in Primary Retroperitoneal Sarcoma: Insights from a Tertiary Cancer Center

**DOI:** 10.3390/cancers17111787

**Published:** 2025-05-27

**Authors:** Carlo Abatini, Lorenzo Barberis, Claudio Lodoli, Federica Ferracci, Enrico De Lorenzis, Giorgio D’Annibale, Matteo Aulicino, Michela Quirino, Mariantonietta Di Salvatore, Sergio Alfieri, Fabio Pacelli, Francesco Santullo

**Affiliations:** 1 Surgical Unit of Peritoneum and Retroperitoneum Surgery, Fondazione Policlinico Universitario Agostino, Gemelli IRCCS, 00168 Rome, Italy; carlo.abatini@policlinicogemelli.it (C.A.); claudio.lodoli@policlinicogemelli.it (C.L.); federica.ferracci@guest.policlinicogemelli.it (F.F.); fabio.pacelli@policlinicogemelli.it (F.P.); francesco.santullo@policlinicogemelli.it (F.S.); 2 General Surgery Department, Università Cattolica del Sacro Cuore, 00168 Rome, Italy; giorgio.dannibale01@icatt.it (G.D.); matteo.aulicino01@icatt.it (M.A.); sergio.alfieri@policlinicogemelli.it (S.A.); 3 Division of Rheumatology, Fondazione Policlinico Universitario Agostino, Gemelli IRCCS, 00168 Rome, Italy; delorenzis.e@gmail.com; 4 Division of Medical Oncology, Fondazione Policlinico Universitario Agostino, Gemelli IRCCS, 00168 Rome, Italy; michela.quirino@policlinicogemelli.it (M.Q.); mariantonietta.disalvatore@policlinicogemelli.it (M.D.S.); 5 Division of Digestive Surgery, Fondazione Policlinico Universitario Agostino, Gemelli IRCCS, 00168 Rome, Italy

**Keywords:** Clavien–Dindo, Sarculator, P-POSSUM

## Abstract

Background: RPS are rare tumors requiring complex surgery. The impact of postoperative complications on long-term outcomes is still debated. This study aimed to identify predictors of severe complications and assess their influence on overall survival (OS) and disease-free survival (DFS). Methods: We retrospectively analyzed 61 patients who underwent curative surgery for primary RPS between 2013 and 2023 at a tertiary cancer center. Patients with pelvic or recurrent sarcomas were excluded. Severe complications were defined as Clavien–Dindo (CD) grade ≥ 3A. Cox regression and Fine–Gray competing risk models were used for OS and DFS, respectively. Sarculator and P-POSSUM scores were included in multivariable models. Results: Severe complications occurred in 14.8% of patients; 30-day mortality was 1.6%. Predictive factors for complications included longer operative time, gastric resection, intraoperative complications, and transfusions. In multivariable analysis, Sarculator was significantly associated with both DFS (HR 0.97; *p* = 0.004) and OS (HR 0.97; *p* = 0.008). The P-POSSUM mortality score predicted OS (HR 1.12; *p* = 0.002). Severe complications were not independently associated with DFS or OS. Conclusions: In this cohort, severe complications did not impact long-term oncological outcomes. Prognostic tools such as Sarculator and P-POSSUM may enhance risk stratification in RPS surgery. Prospective validation is warranted.

## Simple Summary

Patients with retroperitoneal sarcoma (RPS) often require complex surgery, which can lead to serious postoperative complications. It is unclear whether these complications affect long-term outcomes such as survival or cancer recurrence. This study analyzed data from 61 patients treated at a high-volume cancer center to identify which factors predict severe complications and to assess their impact on long-term outcomes. The results showed that although complications were associated with more complex surgeries, they did not independently affect survival or recurrence rates. Two tools used to estimate patient risk, called Sarculator and P-POSSUM, helped predict long-term outcomes more accurately. These findings suggest that better use of risk prediction tools may support clinical decision-making and improve personalized care in retroperitoneal sarcoma surgery.

## 1. Introduction

Retroperitoneal sarcomas (RPS) are rare and heterogeneous malignancies originating from mesenchymal tissue. They represent approximately 10–15% of all soft tissue sarcomas (STS) and about 1% of adult malignancies. In Italy, the incidence of STS is estimated at 5 cases per 100,000 individuals per year, with RPS as a whole comprising a clinically relevant subset [1,2]. Due to their retroperitoneal location and typically indolent growth, RPS often reach substantial size before diagnosis, requiring aggressive and complex surgeries.

Surgical resection with curative intent remains the mainstay of treatment for localized disease. Furthermore, recent evidence supports a histology-driven strategy, tailored to the biological behavior of different sarcoma subtypes [3,4]. For instance, the STRASS trial introduced the rationale for subtype-specific use of neoadjuvant radiotherapy [5]. However, in high-volume tertiary cancer centers, multivisceral resections are frequently required to achieve oncologically adequate margins, particularly for liposarcomas.

Postoperative morbidity following RPS surgery is not uncommon. Major complications are reported in approximately 15–20% of patients, even in experienced centers [6,7]. While some studies suggest that severe complications may adversely impact long-term survival or delay adjuvant treatments, others have found no significant independent association with oncologic outcomes once adjusted for tumor biology and surgical radicality [8,9].

To better stratify risk and guide perioperative decisions, several prognostic models have been developed. Among them, the Sarculator nomogram integrates key clinical and pathological parameters and has been externally validated as a reliable predictor of disease-free survival and overall survival in RPS [10]. Although originally designed for general surgical populations, tools such as the P-POSSUM score have also been investigated specifically for their potential role in oncologic risk estimation [11].

This study aimed to identify the predictors of severe postoperative complications and assess their relationship with long-term outcomes, including overall survival (OS) and disease-free survival (DFS), in a homogeneous cohort of patients with primary RPS. Additionally, we explored the complementary prognostic value of two scoring systems, Sarculator and P-POSSUM, in refining risk stratification and supporting clinical decision-making.

## 2. Materials and Methods

### 2.1. Study Design and Patient Selection

This was a retrospective, single-center cohort study conducted at a tertiary cancer center. We reviewed clinical data from patients who underwent surgical resection for primary retroperitoneal sarcoma (RPS) between January 2013 and December 2023. All procedures were performed with curative intent and employing a histologic subtype-specific strategy: an extended multivisceral resection was performed in all liposarcomas, while a more conservative, but still complete, resection with negative margins was aimed for the other subtypes [12].

Inclusion criteria were as follows: histologically confirmed primary retroperitoneal sarcoma, age ≥ 18 years, and undergoing elective surgery with curative intent. Exclusion criteria included pelvic sarcomas, visceral and mesenteric sarcoma, locally recurrent or metastatic disease at presentation, and patients who received surgery without curative intent. Demographic, clinical, surgical, pathological, and follow-up data were collected from prospectively maintained institutional databases.

### 2.2. Postoperative Complications and Severity Assessment

Postoperative complications occurring within 30 days since hospital admission were evaluated according to the CD classification [13]. Severe complications were defined as grade ≥ 3A, as they require surgical, endoscopic, or radiological interventions under general or local anesthesia. Only the highest-grade complication per patient was considered, in accordance with standard CD methodology [14].

### 2.3. Prognostic Tools

For each patient, two prognostic scores were retrospectively calculated:

- Sarculator: A validated nomogram-based tool that integrates histology, tumor grade, size, multifocality, and completeness of resection to predict 7-year overall survival (OS) and disease-free survival (DFS). The Sarculator has been validated in high-volume centers and recalibrated to reflect recent improvements in outcomes [10].
- P-POSSUM (Portsmouth Physiological and Operative Severity Score for the Enumeration of Mortality and Morbidity): This score combines 12 physiological (age, sex, cardiac signs, systolic blood pressure, pulse rate, Glasgow coma scale, serum urea, serum sodium, serum potassium, hemoglobin, white blood cell count, ECG abnormalities) and 6 surgical parameters (operative severity, number of procedures, blood loss, peritoneal contamination, presence of malignancy, elective or emergency operation) to estimate perioperative morbidity and mortality. Though originally developed for general surgery, it has been explored for oncologic surgery including sarcoma resections [15,16].

### 2.4. Statistical Analysis

Categorical variables were reported as counts and percentages, while continuous variables were summarized as mean ± standard deviation (SD) or median with interquartile range (IQR), depending on the normality of the data, which was assessed by inspection of quantile–quantile plots. Exploratory comparisons between patient groups were conducted using the chi-square test or Fisher’s exact test for categorical variables, as appropriate, and the *t*-test or Mann–Whitney U test for continuous variables, based on the distribution of the data and homogeneity of variances assessed by Levene’s test.

The Kaplan–Meier method was used to estimate a 5-year cumulative mortality after surgery in the overall population and in distinct patient groups. Associations between survival outcomes and postoperative complication severity (according to CDC) or histotype (according to WHO classification) were evaluated using the log-rank test. A Cox proportional hazards model was employed to quantify associations with mortality, with results expressed as hazard ratios (HRs) and 95% confidence intervals (CIs).

A 5-year competing risks survival analysis was also performed. Recurrence-free survival was defined as the time from surgery to cancer recurrence diagnosis. The cumulative incidence function (CIF) was used to account for the competing risk of death. Fine–Gray subdistribution hazard models were applied to estimate sub-hazard ratios (HRs) for recurrence, with the P-POSSUM score as the main predictor. Analyses were adjusted for the occurrence of severe complications (CDC ≥ 3), and cancer severity according to Sarculator score. Results were reported as sub-hazard ratios with 95% confidence intervals.

Statistical significance was set at *p* < 0.05 for all analyses. All tests were two-tailed. Data analysis was performed using RStudio (version 2022.02.3+492).

## 3. Results

### 3.1. Patient Characteristics

A total of 61 patients who underwent surgical resection for primary retroperitoneal sarcoma between 2013 and 2023 were included in the analysis. The median age was 63.2 years (SD ± 11.4), with 60.7% male and 39.3% female. The mean BMI was 26.7 kg/m^2^ (SD ± 4.5). The median Charlson Comorbidity Index (CCI) was 5.0 (IQR 4.0–6.0), and most patients had ECOG performance status 0–1. The majority of tumors were >10 cm in size (mean 215.9 mm, SD ± 132.3), and 73.8% were high-grade (G2–G3) tumors. The predominant histological subtype was liposarcoma (62.3%), including both well-differentiated liposarcomas (WDLPS) and dedifferentiated liposarcomas (DDLPS). Tumors were located on the right side in 54% of cases and on the left in 46%. Overall, complete macroscopic resection (R0/R1) was achieved in 96.7% of patients.

Complete clinical, pathological, and surgical features of the study population are detailed in Table 1.

### 3.2. Postoperative Outcomes and Complications

The median length of hospital stay was 9.0 days (interquartile range [IQR] 6.0–16.0). Overall, 14.8% (9/61) of patients experienced severe postoperative complications (CD grade ≥ 3A), and one patient (1.6%) died within 30 days of surgery.

Details regarding the type and severity of postoperative complications are reported in Table 2 and Table 3, with the most common grade ≥ 3A complications being abdominal collections (*n* = 3), acute bleeding (*n* = 2), and bowel obstruction (*n* = 2).

Patients with severe complications had a significantly higher rate of intraoperative transfusions (66.7% vs. 17.3%, *p* = 0.005), severe intraoperative complications (33.3% vs. 3.8%, *p* = 0.020), and longer mean operative times (398.8 vs. 280.6 min, *p* = 0.048). Gastric resection was significantly more frequent among patients with complications (22.2% vs. 0%, *p* = 0.020). Although both P-POSSUM morbidity and mortality scores were numerically higher in patients with complications, they did not reach statistical significance (*p* = 0.071 and *p* = 0.051, respectively), indicating that P-POSSUM was not a reliable predictor of major complications in this cohort (Table 1). Postoperative complications are reported in Table 2 and Table 3.

### 3.3. Survival and Recurrence Outcomes

Univariate Fine–Gray competing risks regression (Table 4) showed that the Sarculator DFS score was significantly associated with recurrence (HR 0.98, 95% CI 0.96–0.99, *p* = 0.002), while neither severe complications (HR 1.03, *p* = 0.95) nor P-POSSUM mortality score (HR 1.06, *p* = 0.09) were statistically significant. In the multivariate model, Sarculator DFS remained significant (HR 0.97, 95% CI 0.96–0.99, *p* = 0.004), while complications (HR 0.81, *p* = 0.70) and P-POSSUM mortality (HR 1.057, *p* = 0.09) were not.

**Figure 1:**
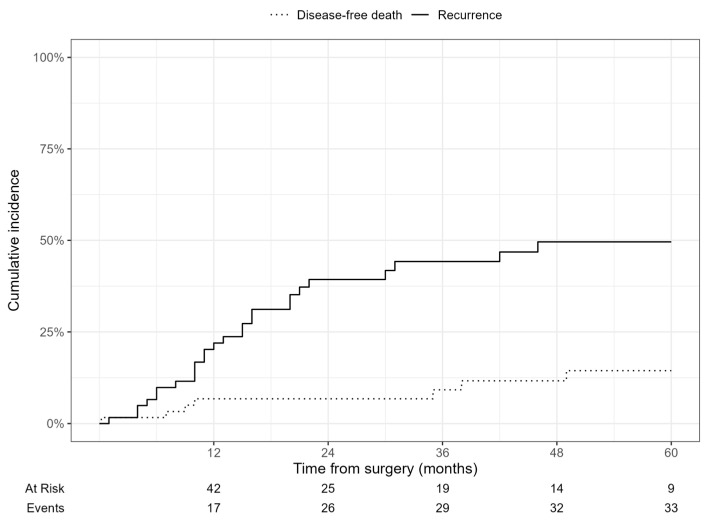
Cumulative incidence of recurrence and disease-free death.

**Figure 2:**
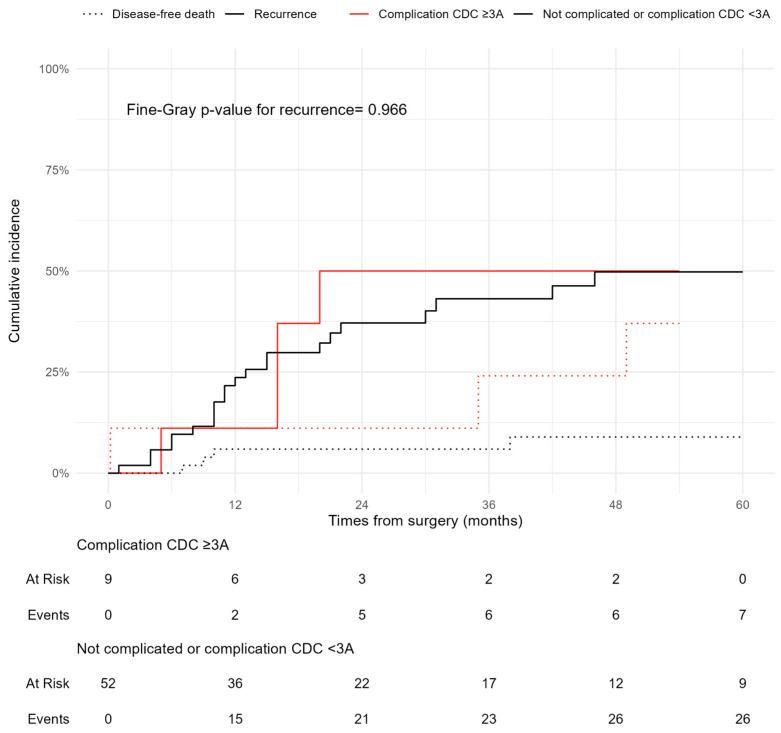
Cumulative Incidence Function (CIF) stratified by complication severity (CD ≥ 3A).

Figure 1 shows the cumulative incidence function (CIF) for recurrence and disease-free death. The 5-year cumulative incidence of recurrence was approximately 50%, while disease-free death occurred in about 20% of patients. Figure 2 stratifies the CIF according to the presence of severe complications (CD ≥ 3A vs. <3A), showing no statistically significant difference (Fine–Gray *p* = 0.966).

### 3.4. Prognostic Factors for Overall Survival

In the univariate Cox model for 5-year overall survival (Table 5), the Sarculator OS score (HR 0.97, 95% CI 0.95–0.99, *p* < 0.001) and P-POSSUM mortality score (HR 1.15, 95% CI 1.08–1.23, *p* < 0.001) were significant predictors, while complications (HR 2.53, *p* = 0.08) showed a trend toward significance. Multivariate analysis confirmed the independent predictive role of Sarculator (HR 0.97, *p* = 0.008) and P-POSSUM mortality (HR 1.12, *p* = 0.002). Severe complications did not independently impact OS in the adjusted model (HR 0.90, *p* = 0.90).

**Figure 3:**
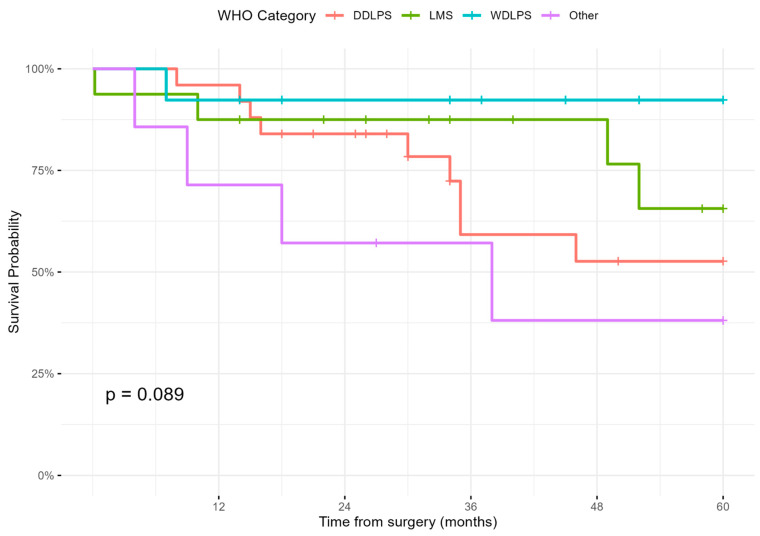
Kaplan–Meier curves by histologic subtype; well-differentiated liposarcoma (WDLPS), dedifferentiated liposarcoma (DDLPS), and leiomyosarcoma (LMS).

**Figure 4:**
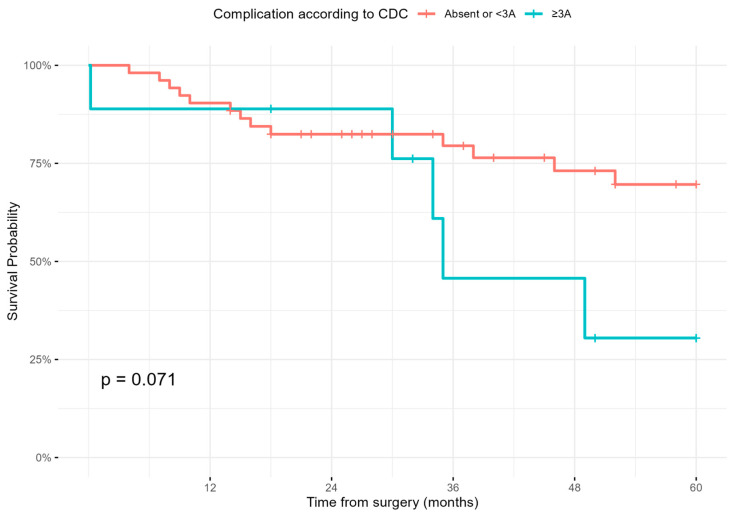
Kaplan–Meier curves by complication severity; Clavien–Dindo classification (CDC).

Kaplan–Meier curves are presented in Figure 3 and Figure 4. Figure 3 stratifies OS by histologic subtype according to WHO classification. Patients with well-differentiated liposarcoma had the most favorable prognosis, while ‘other’ histotypes had the poorest outcomes (*p* = 0.089). Figure 4 shows OS stratified by complication severity, where patients with CDC ≥3A complications demonstrated a trend towards reduced survival (*p* = 0.071).

## 4. Discussion

Postoperative complications remain a pivotal concern in the surgical management of retroperitoneal sarcomas (RPS), as their occurrence can significantly affect short-term outcomes and potentially influence long-term oncologic results. In this study, we aimed to evaluate both the predictors of severe postoperative complications and their prognostic significance on disease-free survival (DFS) and overall survival (OS), using validated tools in a homogeneous patient population.

The incidence of severe complications and 30-day postoperative mortality in our cohort were consistent with those reported by other high-volume sarcoma centers [6,9,17]. Among patients in this series, severe complications were significantly associated with intraoperative transfusions, gastric resections, prolonged operative times, and intraoperative adverse events. These factors have been consistently reported in the literature as proxies for surgical complexity and invasiveness [18,19,20,21].

One-year, three-year, and five-year OS rates in our cohort were 91.8%, 74.8%, and 58.1%, respectively; corresponding DFS rates were 85.1%, 61.2%, and 47.6%. These outcomes are consistent with those reported in both historical and contemporary series of patients undergoing surgery for primary RPS in high- and low-volume institutions [10,22].

Prior studies have demonstrated associations between major postoperative complications and inferior long-term oncologic outcomes [8,23]. A plausible hypothesis is that such complications may activate systemic inflammatory responses and impair functional recovery, thus delaying the initiation of adjuvant therapies, and ultimately increasing recurrence risk. In our analysis, we noted only a non-significant trend toward reduced OS in patients with severe complications (*p* = 0.071). This may reflect the capacity of high-volume tertiary centers to recognize and manage complications promptly and effectively, thereby mitigating their potential long-term impact.

The lack of predictive value of the P-POSSUM score for severe postoperative complications in our cohort is consistent with recent findings [24,25]. In a 2024 study by Angelucci et al., the score was evaluated specifically for its ability to predict short-term morbidity and mortality at 30 days in patients undergoing surgery for soft tissue sarcomas, and its discriminative power was limited [16]. Nonetheless, in our multivariate analysis, the P-POSSUM mortality component was independently associated with OS, suggesting a potential role in broader preoperative risk stratification. One possible explanation for this finding is that P-POSSUM indirectly captures a patient’s physiological reserve and comorbidity burden, which may influence not only perioperative outcomes but also long-term survival as well as prompt access to the most effective chemotherapy regimens. In oncologic surgery, baseline frailty and reduced physiological resilience may impair recovery, limit tolerance to adjuvant therapies, and increase susceptibility to non-cancer-related mortality, thereby affecting long-term prognosis.

The Sarculator nomogram, in contrast, demonstrated robust performance in both univariate and multivariate models for predicting DFS and OS. Its effectiveness likely stems from its integration of multiple clinical and pathological prognostic variables into a validated predictive model [26,27,28]. The concordance between predicted and observed outcomes in our series supports the external validity of the Sarculator and reinforces the reliability of the oncologic results achieved in our cohort.

This study has some noteworthy aspects: It was conducted at a tertiary cancer center, focused on primary retroperitoneal sarcomas, and applied both a validated prognostic model (Sarculator) and an exploratory one (P-POSSUM). Notably, pelvic sarcomas were excluded, due to their distinct anatomical and prognostic characteristics. This distinction is supported by Sarre et al., who emphasized the differential outcome trajectories between pelvic and retroperitoneal sarcomas [29]. Despite these strengths, this study has several limitations, including its retrospective design, and a relatively small sample size, especially for what concerns total number of patients with severe complications (*n* = 9), which may limit the power to detect meaningful associations in multivariable models. Additionally, our study proved to be unable to quantify the cumulative burden of complications using composite metrics such as the Comprehensive Complication Index (CCI). The CCI has been applied also in sarcoma surgery and was proven to offer a more detailed assessment of postoperative morbidity and its clinical impact [30,31]; however, its application in our study was limited by incomplete reporting of minor complications at our institution. In light of this, we started implementing a prospective complication report form for each patient in our surgical ward at discharge.

## 5. Conclusions

Notwithstanding its limitations, this study highlights the incidence and clinical relevance of severe postoperative complications in patients undergoing surgery for primary retroperitoneal sarcoma at a high-volume tertiary cancer center. Although a non-significant trend was observed between complication severity and long-term oncologic outcomes, our findings suggest that the availability of structured multidisciplinary management and timely intervention may mitigate the adverse impact of major complications on overall survival and disease recurrence. In this context, the P-POSSUM mortality score demonstrated independent prognostic value for overall survival, despite not being predictive of severe postoperative complications. These results support its potential utility as an adjunctive tool for preoperative risk stratification and for tailoring perioperative management of high-risk patients. Future prospective studies are needed to validate the integration of multidimensional prognostic tools and morbidity indices, such as the Comprehensive Complication Index, P-POSSUM score, and Sarculator in both clinical decision-making and in the prediction of long-term outcomes in retroperitoneal sarcoma surgery, in order to generate effective composite risk scores.

## Figures and Tables

**Table 1:** Cohort characteristics and associations with severe complications. BMI, Body Mass Index; CCI, Charlson Comorbidity Index; ECOG, Eastern Cooperative Oncology Group; ASA, American Society of Anesthesiologists; P-POSSUM, Portsmouth Physiologic and Operative Severity Score for the Study of Mortality and Morbidity; DDLPS, dedifferentiated liposarcoma; WDLPS, well-differentiated liposarcoma; FNCLCC, Federation Nationale des Centres de Lutte Contre le Cancer.

		Any Complication CD ≥ 3A at 30 Days		
Characteristic	Overall N = 61	NO N = 52	YES N = 9	*p*-Value ^1^
**Age**, Mean ± SD	63.2 ± 11.4	62.9 ± 11.5	65.1 ± 11.7	0.6
**Sex**, *n* (%)				>0.9
Male	37 (60.7%)	31 (59.6%)	6 (66.7%)	-
Female	24 (39.3)	21 (40.4%)	3 (33.3%)	-
**BMI**, Kg/m^2^, mean ± SD	26.7 ± 4.5	26.8 ± 4.7	25.9 ± 3.2	0.6
**CCI**, Median (IQR)	5.0 (4.0, 6.0)	5.0 (4.0, 6.0)	5.0 (3.0, 7.0)	0.6
**ECOG score**, Median (IQR)	1.0 (0.0, 1.0)	1.0 (0.0, 1.0)	1.0 (0.0, 1.0)	0.9
**ASA score**, Median (IQR)	2.0 (2.0, 2.0)	2.0 (2.0, 2.5)	2.0 (2.0, 2.0)	0.7
**P-POSSUM Morbidity**, Median (IQR)	24.7 (17.1, 44.4)	23.4 (17.1, 41.7)	49.1 (31.1, 61.5)	0.071
**P-POSSUM Mortality**, Median (IQR)	4.3 (2.9, 8.7)	4.0 (2.7, 7.5)	10.1 (5.9, 15.4)	0.051
**Disease Location**, *n* (%)				0.3
Right	33 (54%)	30 (57%)	3 (33.3%)	-
Left	28 (46%)	22 (43%)	6 (66.7%)	-
**Histology**, *n* (%)				0.7
Liposarcoma (WDLPS + DDLPS)	38 (62.3%)	33 (63.5%)	5 (55.6%)	-
Other	23 (37.7%)	19 (36.5%)	4 (44.4%)	-
**Tumor Size**, mm, mean ± SD	215.9 ± 132.3	223.4 ± 137.0	172.2 ± 95.6	0.3
**Grading (FNCLCC)**, *n* (%)				0.4
Low Grade (1)	16 (26.2%)	15 (28.8%)	1 (11.1%)	-
High Grade (2–3)	49 (73.8%)	37 (71.2%)	8 (88.9%)	-
**Intraperitoneal Invasion**, *n* (%)				>0.9
Yes	3 (4.9%)	3 (5.7%)	0 (0%)	-
No	58 (95.1%)	49 (94.3%)	9 (100%)	-
**Disease Multifocality**, *n* (%)				0.4
Yes	3 (4.9%)	2 (3.8%)	1 (11.1%)	-
No	58 (95.1%)	50 (69.2%)	8 (88.9%)	-
**Preoperative Chemotherapy**,				0.7
Yes	11 (18.0%)	9 (17.3%)	2 (22.2%)	-
No	50 (82.0%)	43 (82.7%)	7 (77.8%)	-
**Preoperative Radiotherapy**,				>0.9
Yes	4 (6.6%)	4 (7.7%)	0 (0%)	-
No	57 (93.4%)	48 (92.3%)	9 (100%)	-
**Duration of Surgery**, Min, Mean ± SD	298.0 ± 165.9	280.6 ± 146.1	398.8 ± 238.8	0.048
**Completeness of Resection**,				0.3
Complete R0/1	59 (96.7%)	51 (98.1%)	8 (88.9%)	-
Incomplete R2	2 (3.3%)	1 (1.9%)	1 (11.1%)	-
**Number Organ Resected**, Mean ± SD	2.0 (1.0, 3.0)	2.0 (1.0, 3.0)	3.0 (2.0, 4.0)	0.3
**Organ Resected**,				
Small Bowel	2 (3.28%)	2 (3.85%)	0 (0%)	>0.9
Colon	21 (34.4%)	17 (32.7%)	4 (44.4%)	0.7
Stomach	2 (3.28%)	0 (0%)	2 (22.2%)	0.020
Pancreas	3 (4.92%)	2 (3.85%)	1 (11.1%)	0.4
Spleen	4 (6.56%)	3 (5.77%)	1 (11.1%)	0.5
Diaphragm	4 (6.56%)	2 (3.85%)	2 (22.2%)	0.10
Nerve	3 (4.92%)	2 (3.85%)	1 (11.1%)	0.4
Psoas	15 (24.6%)	12 (23.1%)	3 (33.3%)	0.7
Vascular	8 (13.1%)	7 (13.5%)	1 (11.1%)	>0.9
Kidney + Adrenal gland	30 (49.2%)	26 (50.0%)	4 (44.4%)	>0.9
Uterus + Adnexa	3 (4.92%)	3 (5.77%)	0 (0%)	>0.9
Other	6 (9.84%)	5 (9.62%)	1 (11.1%)	>0.9
**Intraoperative Transfusion**,				0.005
Yes	15 (24.6%)	9 (17.3%)	6 (66.7%)	-
No	46 (75.4%)	43 (82.7%)	3 (33.3%)	-
**Severe Intraoperative Complication**,				0.020
Yes	5 (8.2%)	2 (3.8%)	3 (33.3%)	-
No	56 (91.8%)	50 (96.2%)	6 (66.7%)	-

^1.^ Two Sample *t*-test; Fisher’s exact test; Wilcoxon rank sum test.

**Table 2:** Postoperative complications.

Factor	N (%)
**Any complications**, *n* (%)	
No	37 (60.7%)
Yes	24 (39.3%)
**Number of complications per patient**, *n* (%)	
0	38 (62.3%)
1	19 (31.1%)
2	3 (4.92%)
3	1 (1.64%)
**Patient with complication CD ≥ 3A**, *n* (%)	
No	52 (85.2%)
Yes	9 (14.8%)

**Table 3:** Details of complications CD ≥ 3A.

Type	Grade	N.
Acute bleeding	3B	2
	5	1
Abdominal Collection	3A	3
Bowel obstruction	3B	2
Urinary fistula	3A	1
		Tot 9

**Table 4:** Disease-free survival at 5 years (Fine–Gray regression).

	Univariate			Multivariate		
	HR	95% CI	*p*-Value	HR	95% CI	*p*-Value
**Complication CD ≥ 3A**	1.03	0.37–2.87	0.95	0.81	0.28–2.33	0.7
**Sarculator DFS**	0.98	0.96–0.99	0.002	0.97	0.96–0.99	0.004
**P-Possum score mortality**	1.06	0.99–1.14	0.09	1.057	0.99–1.12	0.09

**Table 5:** Mortality at 5 years (Cox regression).

	Univariate			Multivariate		
	HR	95% CI	*p*-Value	HR	95% CI	*p*-Value
Complication CD ≥ 3A	2.53	0.90–7.41	0.08	0.90	0.27–2.04	0.9
Sarculator OS	0.97	0.95–0.99	<0.001	0.97	0.95–0.99	0.008
P-Possum score mortality	1.15	1.08–1.23	<0.001	1.12	1.04–1.21	0.002

## Data Availability

The raw data supporting the conclusions of this article will be made available by the authors on request.
